# Throat Disease of Public Speakers

**Published:** 1843-01

**Authors:** 


### THROAT DISEASE OF PUBLIC SPEAKERS.

At the annual meeting of the Medical Convention, of Ohio, at Cincinnati, in the month of May last, a committee consisting of Dr.

Bœrstler, of Lancaster; Dr. Sams, of Hillsborough; Dr. Fisher, of Waynsville; Dr. Warder, of Cincinnati; and the writer of this notice, Dr. Drake; was appointed to "inquire into, and report to the next Convention, on the causes and prevention of the *chronic laryngitis* of clergymen, and other public speakers."

Communications from physicians and other gentlemen, are solicited, and may be made to either member of the committee. For the purpose of directing the course of inquiry, of all who may feel able or disposed to advance it, we present the following interrogative suggestions:

1. Is not the fauces, or visible parts of the throat inflamed? And may not this inflammation sometimes precede that of the larynx which produces the flat and husky voice?

2. Is not the inflammation of the throat, in some cases, connected with a dyspeptic state of the stomach?

3. Is it not in others, associated, as a sympathetic affection, with chronic disease of the lungs, or of the lungs and heart?

4. Has it increased, with the decline of the use of alcoholic drinks, more or less resorted to, in former times, to allay the irritation, and remove the feeling of debility in the throat, *immediately* after speaking?

5. Is there any connexion of cause and effect, between the disuse of tobacco and the greater frequency of this malady, as suggested by Professor Mott?

6. Is it more frequent where coal is the principal fuel, than where wood is burnt? Do clergymen and other studious persons who sit near stoves, keep up evaporation from *non*-metallic pans, so shallow as to secure a copious humidity?

7. Are itinerant clergymen, and those who perform manual labor, as liable to the disease as the sedentary?

8. Are the Episcopal clergy, who read the liturgy of that church with studied inflexions of voice, and generally read their sermons, more subject to the disease than those who read but little in public, and extemporise their sermons?

9. Is the disease limited to the middle latitudes of the Union, or does it attack those who live on the borders of the Gulf and the Lakes?

10. Is it possible to arrest the absurd application of the term *bronchitis*, signifying an inflammation of the air tubes and cells of the lungs, to this affection of the organ of voice—the *larynx?*

We respectfully ask of newspaper editors, who may chance to meet with this article to extend its publicity. We would, also, solicit the secretaries of our different ecclesiastical conventions, conferences and assemblies, to have it read during their sessions of the present year, as a means of securing the attention of the class of men most interested in this investigation. We shall rely on our brethren of the medical press, to make it known to the profession beyond the limits of our circulation. D.

